# Clinical Severity and Surgical Burden in Drug Use-Associated Infective Endocarditis: A Six-Year Cohort Study

**DOI:** 10.3390/microorganisms14010111

**Published:** 2026-01-05

**Authors:** Corina-Ioana Anton, Bogdan Mircea Petrescu, Cosmin Alexandru Buzilă, Ion Ștefan, Cristian Sorin Sima, Adrian Streinu-Cercel

**Affiliations:** 1 Department of Infectious Diseases, “Dr. Carol Davila” Central Military Emergency University Hospital, 134 Calea Plevnei, 010242 Bucharest, Romania; corina-ioana.anton@drd.umfcd.ro (C.-I.A.); dr.stefanion@gmail.com (I.Ș.); 2 Department of Medico-Surgical and Prophylactic Disciplines, Titu Maiorescu University, 040441 Bucharest, Romania; 3 Faculty of General Medicine, Carol Davila University of Medicine and Pharmacy, 8 Eroii Sanitari Bvd., 050474 Bucharest, Romania; cristian.sima@drd.umfcd.ro (C.S.S.); adrian.streinucercel@umfcd.ro (A.S.-C.); 4 Department of Clinical Psychiatry, “Dr. Carol Davila” Central Military Emergency University Hospital, 134 Calea Plevnei, 010242 Bucharest, Romania; 5 Cardiovascular Surgery Department, “Dr. Carol Davila” Central Military Emergency University Hospital, 134 Calea Plevnei, 010242 Bucharest, Romania; buzilacosmin@yahoo.com; 6 Department of Infectious Diseases I, Faculty of Medicine, Carol Davila University of Medicine and Pharmacy, 020021 Bucharest, Romania; 7 National Institute for Infectious Diseases “Prof. Dr. Matei Balş”, 1 Dr. Calistrat Grozovici Street, 021105 Bucharest, Romania

**Keywords:** infective endocarditis, injection drug use, opioid use disorder, valve surgery, *Staphylococcus aureus*

## Abstract

Drug use–associated infective endocarditis (DUA-IE) is an increasingly important clinical problem that affects younger patients and poses substantial diagnostic, therapeutic, and surgical challenges. We conducted a retrospective cohort study of adults with definite infective endocarditis treated at a tertiary referral center between 2017 and 2022, comparing patients with DUA-IE to those with non–drug use–associated infective endocarditis. Of the 189 patients, 43 (22.8%) had DUA-IE. These patients were significantly younger and had higher rates of HIV and hepatitis C coinfections. *Staphylococcus aureus* was the predominant pathogen, and right-sided valve involvement was more frequent; however, left-sided disease predominated among patients requiring valve surgery. Compared with non-DUA-IE patients, those with DUA-IE had larger vegetations, higher inflammatory markers, more frequent complications(including sepsis, embolic events, and heart failure), higher rates of emergency surgical intervention, longer hospitalizations, and increased in-hospital mortality rates. In conclusion, DUA-IE represents a distinct and more aggressive form of infective endocarditis, characterized by severe infection, increased complication rates, and a substantial surgical burden despite the younger patient age, underscoring the need for integrated infectious disease, surgical, and addiction-focused care models for these patients.

## 1. Introduction

Infective endocarditis (IE) remains a severe and potentially life-threatening disease associated with high morbidity, mortality, and healthcare resource utilization, despite advances in diagnostic imaging, antimicrobial therapy, and cardiac surgery [1]. Global epidemiological data indicate a rising incidence of IE in recent decades, reflecting changes in population demographics, healthcare exposure, and risk factor profiles [1]. This evolving epidemiology has prompted renewed interest in identifying distinct IE subgroups with unique clinical and management challenges [2].

One of the most important drivers of this epidemiological shift is drug use–associated infective endocarditis (DUA-IE) [2]. DUA-IE predominantly affects younger patients and is frequently accompanied by complex medical and social vulnerabilities, including viral coinfections, psychiatric disorders, and unstable access to healthcare [1,2]. From a microbiological perspective, drug use-related infections are commonly caused by highly virulent pathogens, most notably *Staphylococcus aureus*, reflecting direct bloodstream inoculation during injection practices [3]. These characteristics distinguish DUA-IE from more traditional forms of IE associated with degenerative valve disease, healthcare exposure, and prosthetic materials [3].

The clinical management of DUA-IE is particularly challenging. In addition to infection-related factors, clinicians must address issues related to adherence to prolonged antimicrobial therapy, frequent discharge against medical advice, and complex decision-making regarding surgical interventions [3]. While right-sided valve involvement is classically associated with injection drug use, both right- and left-sided diseases may occur, and the factors driving surgical referral in this population remain incompletely characterized [4]. Moreover, data describing the interaction between clinical presentation, microbiology, echocardiographic findings, surgical burden, and short-term outcomes in DUA-IE are limited, particularly in Central and Eastern Europe [5,6].

To address these gaps, we conducted a six-year retrospective cohort study of adults with definite IE who were treated at a tertiary referral center. The primary objective was to compare patients with DUA-IE and non-DUA-IE with respect to demographic characteristics, comorbidities, microbiological profiles, echocardiographic findings, need for surgical intervention, and in-hospital outcomes. By delineating the distinctive features of DUA-IE within a single healthcare system, this study aims to contribute clinically relevant data to inform the diagnostic, therapeutic, and surgical strategies for this vulnerable population.

## 2. Materials and Methods

### 2.1. Study Design and Setting

We performed a retrospective cohort study using clinical, microbiological, echocardiographic, and surgical data collected between 2017 and 2022 at the “Dr. Carol Davila” Central Military Emergency University Hospital in Bucharest, Romania. This tertiary care institution includes both an infectious diseases department and a cardiovascular surgery department, providing comprehensive management for complex cases of IE, including those requiring surgical intervention.

### 2.2. Study Population and Definitions

We screened the hospital discharge database for all adult patients (≥18 years) hospitalized with a diagnosis of infective endocarditis between 1 January 2017, and 31 December 2022. Definite IE was defined according to the modified Duke criteria (two major criteria, or one major plus three minor criteria, or five minor criteria). Major criteria were based on typical blood culture results and echocardiographic evidence of endocardial involvement. Minor criteria included predisposing conditions (including drug use disorder), fever ≥38 °C, vascular phenomena, immunological phenomena, and microbiological evidence not meeting major criteria.

Patients with native-valve IE, prosthetic-valve IE, and first-episode IE were included. Recurrent IE episodes occurring during the study period were excluded.

Patients were classified into two groups as follows:

• DUA-IE group: IE in patients with a documented history of drug use disorder (including injection and non-injection routes), as recorded in the medical files. Non-injection drug use primarily included inhalational or intranasal opioid or stimulant use documented in the medical records; however, in most cases, injection drug use was either current or previously reported.
• Non-DUA-IE group (control group): IE in patients with no documented drug use disorder.
• Patients younger than 18 years and those with incomplete data were excluded.

### 2.3. Data Collection

Data were extracted from electronic and paper medical records using a standardized form and included the following:

• Demographics: age, sex
• Clinical presentation: symptoms and hemodynamic status
• Comorbidities: HIV, HCV, HBV, hepatocellular carcinoma, chronic renal failure, hypertension, chronic alcoholism, prior stroke, and others
• Hematochemical parameters at admission: white blood cell count, C-reactive protein, creatinine, and hemoglobin levels
• Echocardiographic findings: affected valves, presence and size of vegetation, abscesses, and degree of regurgitation
• Microbiology: blood culture results and identified pathogens
• Complications during hospitalization: persistent bacteremia (>72 h), sepsis, septic shock, embolic events (pulmonary or systemic), and heart failure
• Surgical data: indication for surgery, valve(s) operated on, type of valve prosthesis, and postoperative complications
• Outcomes: length of hospital stay, discharge destination (home, rehabilitation, nursing facility), discharge against medical advice, and in-hospital mortality.

### 2.4. Microbiological Methods

Blood cultures were obtained from all patients as part of the standard diagnostic workup for IE. Two sets of blood specimens were drawn from different venous sites, each consisting of aerobic and anaerobic bottles. The specimens were processed using the BACTEC automated blood culture system. Positive cultures were subcultured, and organisms were identified using the VITEK 2 automated system. Antimicrobial susceptibility testing was performed using disk diffusion and interpreted according to CLSI standards [7]

### 2.5. Echocardiographic Assessment

Transthoracic echocardiography (TTE) was performed in all patients at the time of diagnosis as part of the standard evaluation for suspected infective endocarditis. Transesophageal echocardiography (TEE) was performed in cases with inconclusive TTE findings, suspected prosthetic valve endocarditis, suspected periannular extension, or when intracardiac complications were clinically suspected. Although current ESC guidelines recommend routine TEE in all patients with suspected IE, in real-world clinical practice TEE was not feasible in a minority of cases due to patient instability, contraindications, or refusal; however, all included patients fulfilled the modified Duke criteria for definite infective endocarditis based on combined clinical, microbiological, and imaging findings.

Advanced cardiac imaging modalities, including cardiac computed tomography (CT), magnetic resonance imaging (MRI), positron emission tomography (PET/CT), or single-photon emission computed tomography (SPECT), were not routinely used during the study period and were reserved for selected cases based on clinical judgment and local availability. Screening for extracardiac complications was performed using targeted imaging studies (brain CT or MRI, thoracic CT, or abdominal CT) in patients with neurological symptoms, suspected embolic phenomena, persistent bacteremia, or signs of systemic infection, and musculoskeletal CT or MRI in patients with focal osteoarticular symptoms suggestive of septic arthritis/osteomyelitis (including sacroiliitis).

The following data were collected:

• Presence and location of vegetation
• Vegetation size (largest diameter measured in mm)
• Valve involvement (aortic, mitral, tricuspid, and pulmonic);
• Presence of abscesses, pseudoaneurysms, or leaflet perforation
• Degree of valvular regurgitation (mild, moderate, or severe);
• Left and right ventricular functions were reported.

Valve involvement was categorized as native-valve IE or prosthetic-valve IE based on the presence of a surgically implanted valve at the time of diagnosis. Valve location was classified as left-sided (aortic and/or mitral valve involvement), right-sided (tricuspid and/or pulmonic valve involvement), or combined left- and right-sided disease. In cases with multivalvular involvement, patients were classified according to all affected valves.

### 2.6. Definitions of Complications

All patients were systematically evaluated for IE–related complications during hospitalization. Clinical assessment was performed daily by the treating team, and targeted diagnostic investigations were undertaken based on clinical suspicion or persistence of infection.

Neurological complications, including ischemic or hemorrhagic stroke, were evaluated using brain computed tomography (CT) or magnetic resonance imaging (MRI) in patients presenting with focal neurological deficits, altered mental status, or embolic suspicion. Embolic events were confirmed using appropriate imaging modalities, including thoracic CT for pulmonary emboli and abdominal CT for splenic or renal infarctions. Musculoskeletal infectious complications (e.g., sacroiliitis) were evaluated using targeted CT or MRI when clinically suspected.

Heart failure was diagnosed based on clinical criteria supported by echocardiographic evidence of severe valvular regurgitation or ventricular dysfunction. Periannular complications, including abscess formation, pseudoaneurysm, valve perforation, or dehiscence, were identified using transthoracic and transesophageal echocardiography.

Persistent bacteremia was defined as at least one positive blood culture obtained ≥72 h after initiation of appropriate antimicrobial therapy. Sepsis and septic shock were defined according to the Sepsis-3 criteria [8].

### 2.7. Surgical Management and Indications

Surgical indications followed the European Society of Cardiology (ESC) guidelines and included the following:

• Acute severe valvular regurgitation causing heart failure
• Uncontrolled infection despite adequate antibiotic therapy
• Periannular complications, such as abscesses or fistulas
• Large vegetations (>10 mm) with recurrent emboli or a very high embolic risk
• Prosthetic valve infection with structural deterioration.

The type of surgery (valve repair vs. replacement), valve location, and prosthesis type (mechanical vs. biological) were also recorded.

### 2.8. Antimicrobial Therapy

Empiric antimicrobial therapy was initiated promptly after blood cultures were obtained, in accordance with contemporary European Society of Cardiology (ESC) and national guidelines for the management of infective endocarditis. Empiric regimens were selected based on clinical severity, suspected valve involvement (native vs. prosthetic), and local microbiological epidemiology, with particular attention to coverage of *S. aureus*, streptococci, and enterococci.

Initial empiric therapy most commonly consisted of a β-lactam antibiotic (e.g., ceftriaxone, ampicillin–sulbactam, or flucloxacillin) in combination with a glycopeptide (vancomycin), with or without an aminoglycoside, depending on renal function and hemodynamic stability. Following microbiological identification and antimicrobial susceptibility testing, antibiotic regimens were de-escalated or modified to targeted therapy in accordance with guideline recommendations.

All patients received intravenous antibiotic therapy during hospitalization. The standard duration of treatment was six weeks for native-valve infective endocarditis and eight weeks for prosthetic-valve infective endocarditis, with adjustments made in cases of periannular complications, persistent bacteremia, or surgical intervention. Antibiotic therapy was continued postoperatively when indicated, with duration calculated from the date of valve surgery in surgically treated patients.

Outpatient parenteral antibiotic therapy (OPAT) was not routinely implemented during the study period. In patients with drug–use–associated infective endocarditis, prolonged inpatient treatment was generally preferred due to concerns regarding treatment adherence, ongoing substance use, and limited outpatient monitoring infrastructure. Selected clinically stable patients without ongoing drug use were discharged with continued antimicrobial therapy under close infectious disease follow-up.

### 2.9. Statistical Analysis

Continuous variables were summarized as medians with interquartile ranges or means with standard deviations, as appropriate. Categorical variables were expressed as absolute numbers and percentages. Between-group comparisons were performed using the Mann–Whitney U test or Student’s *t*-test for continuous variables and the χ^2^ test or Fisher’s exact test for categorical variables.

Given the number of microbiological comparisons performed, isolated statistically significant differences with small absolute frequencies should be interpreted with caution and considered exploratory.

Given the exploratory and descriptive nature of this retrospective cohort study, *p*-values were interpreted descriptively, and no formal correction for multiple testing was applied. Consequently, statistically significant *p*-values should be viewed as hypothesis-generating rather than confirmatory. Statistical analyses were performed using SPSS version 26.

### 2.10. Ethics

The study protocol was approved by the Ethics Committee of the “Dr. Carol Davila” Central Military Emergency University Hospital (Decision No. 562/20.12.2022).

## 3. Results

### 3.1. Overall Cohort and Baseline Characteristics

**Figure 1:**
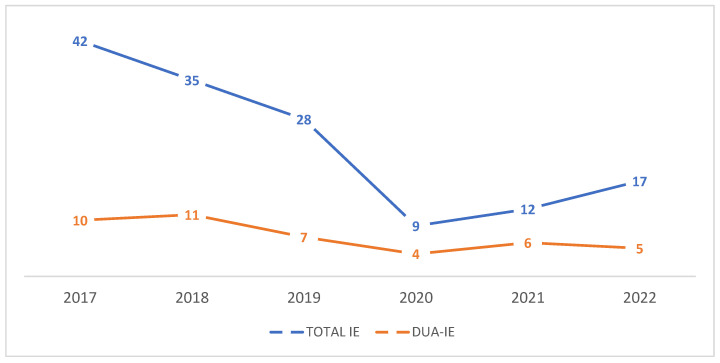
Annual number of IE and DUA-IE cases recorded between 2017 and 2022 at the “Dr. Carol Davila” Central Military Emergency University Hospital, Bucharest.

Between 2017 and 2022, 189 adult patients met the inclusion criteria. Among these, 43 (22.8%) were classified as DUA-IE and 146 (77.2%) as non-DUA-IE. Annual trends in total IE and DUA-IE cases from 2017 to 2022 are shown in Figure 1.

DUA-IE patients were markedly younger, with a median age of 36 years (interquartile range [IQR] 24–41), compared with 62 years (IQR 53–89) in non-DUA-IE patients. Men predominated in both groups, but the male:female distribution was similar, suggesting that sex alone did not distinguish drug use–associated IE from other forms of IE (Table 1).

### 3.2. Hematochemical Parameters

Hematochemical data (Table 2) revealed more intense inflammatory responses in the DUA-IE group. DUA-IE patients had higher mean white blood cell counts and C-reactive protein levels than non-DUA-IE patients, indicating more severe systemic inflammation at presentation. Creatinine levels were slightly higher and hemoglobin levels slightly lower in the DUA-IE group; however, these differences were not statistically significant.

These findings suggest that, despite being younger and ostensibly less burdened by chronic cardiovascular disease, patients with DUA-IE tend to present with more aggressive infections and more severe inflammatory responses.

### 3.3. Microbiological Findings

Blood cultures were positive in all patients with DUA-IE. *Staphylococcus aureus* emerged as the predominant pathogen in the DUA-IE group, responsible for approximately 55.9% of cases, compared with 43.1% in the non-DUA-IE group. *Enterococcus faecalis* and *Streptococcus mitis* were also frequent, particularly in non-DUA-IE cases. Gram-negative organisms, such as *Klebsiella pneumoniae,* were rare but occurred exclusively in DUA-IE. (Table 3).

Classical viridans group streptococci were infrequently identified in this cohort, likely reflecting changing epidemiology, widespread antibiotic exposure prior to hospitalization, and the predominance of staphylococcal infections; notably, no cases of culture-negative infective endocarditis were recorded in the DUA-IE group.

This microbiological profile is consistent with injection-related infections, where skin flora, especially *S. aureus*, are directly inoculated into the bloodstream. The higher proportion of *S. aureus* among DUA-IE cases likely contributes to the more severe clinical course and higher rate of complications observed in this group.

### 3.4. Echocardiographic Findings and Valve Involvement

Among patients with DUA-IE, IE predominantly involved native valves, with prosthetic-valve infection occurring in a minority of cases. Right-sided valve involvement, particularly of the tricuspid valve, was more frequent in DUA-IE, whereas non-DUA-IE patients more commonly presented with left-sided disease involving the aortic and mitral valves. Combined left- and right-sided involvement was uncommon in both groups but occurred more frequently in patients with extensive disease and complications.

Echocardiography identified vegetations in most patients in both groups. As shown in Table 4, median vegetation size was significantly larger in patients with DUA-IE compared with non–DUA-IE patients. Severe tricuspid regurgitation was significantly more frequent in the DUA-IE group, whereas severe aortic and mitral regurgitation occurred at slightly similar rates in both groups.

Due to the descriptive nature of echocardiographic severity indicators and overlapping clinical features, formal statistical comparison was not performed for all parameters. Echocardiographic severity indicators further differentiated the two groups. Median vegetation size was larger in DUA-IE patients (16 mm, IQR 12–22 mm) compared with non-DUA-IE patients (12 mm, IQR 8–17 mm), supporting the observation that injection-related infections tend to present with bulkier and more mobile vegetations.

However, a different pattern emerged when focusing on patients who underwent valve surgery. Among the 83 patients (43.9% of the cohort) who required valve surgery, 28 (33.7%) belonged to the DUA-IE group and 55 (66.3%) to the non-DUA group. In these surgically treated DUA-IE patients, aortic valve replacement was performed in 64.2% of cases, mitral valve replacement in 25% of cases, and tricuspid valve replacement in only 10.7% of cases. In the non-DUA-IE surgical group, mitral valve replacement was slightly more prevalent than aortic valve replacement, and tricuspid replacement was relatively uncommon.

At first glance, this surgical valve distribution appears to contradict the classical epidemiology of DUA-IE, which emphasizes tricuspid valve involvement. However, this apparent discrepancy is best explained by surgical selection bias.

Isolated right-sided (tricuspid) IE, particularly in people who inject drugs, often responds well to medical therapy alone. Left-sided IE (aortic or mitral) is associated with severe regurgitation, heart failure, persistent bacteremia, and systemic embolization, which more frequently necessitate surgery. Consequently, although tricuspid valve IE may be more common overall in DUA-IE, left-sided disease is overrepresented among surgical candidates.

Thus, our findings are consistent with the broader experience that surgical cohorts of DUA-IE may predominantly feature left-sided valve operations, even in populations in which right-sided involvement is epidemiologically more common.

### 3.5. Surgical Indications and Procedures

In total, 83 patients underwent cardiac surgery. Aortic valve replacement was the most frequent procedure in the DUA-IE group (64.2%), followed by mitral valve replacement (25.0%), while tricuspid valve replacement accounted for only 10.7% of surgeries. Conversely, among non-DUA-IE patients, tricuspid valve replacement was performed in 23.6% of cases. (Table 5) This detailed distribution underscores that the majority of operations addressed left-sided valve pathology, even in the DUA-IE population.

Although right-sided valve involvement was common in DUA-IE, surgical intervention was predominantly required for left-sided native-valve disease due to severe valvular dysfunction, persistent infection, or embolic risk.

Several surgical techniques were used depending on the extent of valvular destruction, including complete removal of infected tissue, annular reconstruction and replacement with either mechanical or bioprosthetic valves. Tricuspid procedures involved the excision of large vegetations, debridement of necrotic leaflets and valve replacement when repair was not feasible. Left-sided interventions frequently required extensive debridement of periannular abscesses and in some cases aortic root reconstruction, reflecting the severity and complexity of disease at the time of surgical evaluation.

In the DUA-IE group, surgery was often prompted by complex clinical scenarios combining large vegetations, ongoing sepsis, and heart failure. Most interventions involved valve replacement rather than repair, reflecting the extent of structural damage at the time of diagnosis. Both mechanical and biological prostheses were used; however, in younger DUA-IE patients, the choice of prosthesis had to balance durability against the risk of reinfection and the feasibility of long-term anticoagulation.

Postoperative complications included recurrent infection, stroke, and prolonged need for intensive care in some cases. Nonetheless, surgical intervention offers the only realistic chance of survival for patients with advanced left-sided disease.

### 3.6. Comorbidities and Coinfections

The comorbidity patterns differed significantly between the groups. Patients with DUA-IE had much higher rates of HIV infection (65.1%), AIDS, hepatitis C, and hepatitis B. Chronic alcoholism and cellulitis were also significantly more frequent. In contrast, non-DUA-IE patients had a higher prevalence of chronic renal failure, hypertension, and prior stroke, reflecting a more traditional cardiovascular risk profile.

These differences underscore the need for integrated care addressing both infectious comorbidities and substance use, particularly among patients with DUA-IE.

Comorbidities revealed a striking contrast between the two groups (Table 6): DUA-IE patients had a significantly higher prevalence of infectious comorbidities, including HIV (65.1% vs. 1.37%), hepatitis C (25.5% vs. 7.5%), and hepatitis B (4.6% vs. 0), as well as chronic alcoholism and cellulitis. In contrast, non-DUA-IE patients had a higher burden of hypertension (53.4%) and prior stroke (27.3%), consistent with an older, more comorbid, cardiovascular population.

### 3.7. Infective Endocarditis–Related Complications and Clinical Outcomes

Patients with DUA-IE experienced a significantly higher burden of infective endocarditis-related complications compared with non-DUA-IE patients. The most frequently observed complications included persistent bacteremia, sepsis, embolic events (pulmonary and systemic), heart failure due to severe valvular dysfunction, and periannular extension such as abscess formation or leaflet perforation.

A comparative summary of IE–related complications and in-hospital outcomes according to drug use status is presented in Table 7.

As summarized in Table 7, patients with DUA-IE experienced significantly higher rates of emergency valve surgery and discharge against medical advice, as well as longer hospital stays, compared with non–DUA-IE patients. In-hospital mortality was also significantly higher in the DUA-IE group. Median vegetation size was significantly larger in DUA-IE, whereas periannular complications and severe valvular regurgitation were more frequent but did not differ significantly between groups. Stroke occurred more frequently in non–DUA-IE patients.

Complications were defined as described in Section 2.6. Emergency valve surgery refers to surgical intervention performed during the index hospitalization due to heart failure, uncontrolled infection, or high embolic risk.

Persistent bacteremia and sepsis were significantly more common, consistent with the predominance of *S. aureus* and large vegetations. Embolic events, both pulmonary and systemic, frequently occurred in DUA-IE, reflecting the high embolic potential of large, mobile vegetation.

Hospital stays were longer in DUA-IE, with median durations significantly exceeding those in non-DUA-IE, particularly among surgically treated patients, where postoperative recovery and management of comorbidities required extended hospitalization. (Table 8).

Neurological complications, including ischemic stroke, were more frequently observed in patients with non-DUA-IE, reflecting their older age and higher prevalence of pre-existing cerebrovascular disease. In contrast, patients with DUA-IE more commonly developed septic and embolic complications, particularly pulmonary emboli associated with right-sided valve involvement. Heart failure and severe valvular insufficiency were common indications for surgical intervention in both groups but occurred earlier and more frequently in DUA-IE due to aggressive valvular destruction.

Outcome data highlighted the substantial challenges in managing DUA-IE. Patients with DUA-IE were less likely to be discharged home and more likely to leave against medical advice. In-hospital mortality was higher in DUA-IE than in non-DUA-IE, despite the younger age of patients. The combination of aggressive infection, complex comorbidities, social vulnerability, and incomplete treatment courses likely contributes to these poor outcomes.

### 3.8. Antibiotic Treatment and Susceptibility

Empiric antibiotic regimens were broadly similar in the two groups and were initiated promptly after blood cultures were obtained. Most patients received a combination of a β-lactam and a glycopeptide, with or without an aminoglycoside, reflecting the high prevalence of *S. aureus* and *Enterococcus* species in this cohort. Targeted therapy was subsequently adjusted in accordance with susceptibility testing.

Among *S. aureus* isolates, 74.3% were methicillin-susceptible (MSSA) and 25.7% methicillin-resistant (MRSA) in the DUA-IE group, compared with 44.1% MSSA and 55.9% MRSA in the non-DUA-IE group. All MSSA strains remained susceptible to oxacillin/nafcillin and cefazolin, whereas MRSA isolates were uniformly susceptible to vancomycin and linezolid, and 11.6% showed reduced susceptibility or resistance to clindamycin.

*Enterococcus faecalis* isolates were susceptible to ampicillin in 72.4% of DUA-IE cases and of 81.3% non-DUA-IE cases, with high-level aminoglycoside resistance detected in 47.2% and 41.3%, respectively. *Streptococcus mitis* and other viridans streptococci were generally susceptible to β-lactams, and no clinically relevant resistance patterns were observed. Gram-negative organisms, which occurred exclusively in DUA-IE (*Klebsiella pneumoniae*), were susceptible to third-generation cephalosporins in 73.6% of isolates, with 62.8% demonstrating extended-spectrum β-lactamase production.

Overall, there were no major differences in susceptibility patterns between DUA-IE and non-DUA-IE, although DUA-IE tended to show slightly higher rates of methicillin resistance among *S. aureus* isolates and a marginally greater prevalence of high-level aminoglycoside resistance in *Enterococcus faecalis*. These findings are consistent with the microbiological profile dominated by *S. aureus* and *Enterococcus* species and underscore the importance of early, appropriately broad empiric coverage in patients with suspected DUA-IE.

Daptomycin was not routinely used during the study period and was reserved for selected salvage cases; therefore, it was not included among standard empiric or first-line targeted regimens.

All patients in both groups received empiric intravenous antibiotic therapy followed by targeted treatment based on blood culture identification and antimicrobial susceptibility testing. There were no relevant differences between DUA-IE and non-DUA-IE patients regarding initial empiric antibiotic regimens. However, patients with DUA-IE more frequently required prolonged inpatient intravenous therapy due to persistent bacteremia, complications, or social and behavioral factors that limited eligibility for outpatient parenteral antibiotic therapy. Outpatient intravenous antibiotic treatment was used selectively and was uncommon in patients with active drug use.

## 4. Discussion

This study provides an extensive characterization of DUA-IE compared to non-DUA-IE in a large Romanian tertiary center over a six-year period. The findings demonstrate differences in epidemiology, comorbidities, microbiology, disease severity, surgical involvement, and outcomes between the two groups, underscoring the unique and multifaceted challenges associated with managing endocarditis among people who inject drugs.

One of the most striking observations was the demographic distribution of the cohort. Patients with DUA-IE were markedly younger than those with non-DUA-IE, often by nearly three decades, confirming global trends that injection drug use increasingly drives IE in younger populations compared to older populations. Despite this substantial age difference, the male-to-female distribution was similar across groups, suggesting that sex is not a distinguishing factor in this population. However, the younger age profile of DUA-IE patients did not translate into more favorable clinical outcomes, highlighting the profound impact of comorbidities and infection severity.

The results clearly demonstrate that DUA-IE is associated with a distinct comorbidity profile dominated by infectious diseases. As shown in Table 5, the prevalence of HIV, AIDS, hepatitis C, and hepatitis B was dramatically higher in the DUA-IE group. Concurrent dermatologic infections, such as cellulitis, chronic alcoholism, and other markers of social vulnerability further characterize this high-risk group. In contrast, non-DUA-IE patients displayed a more typical cardiovascular risk profile, including hypertension, chronic renal disease, and previous cerebrovascular events. These comorbidity patterns substantially shape the disease trajectory and management complexity, reinforcing the need for integrated infectious disease, addiction, and social care in this population [9].

Laboratory parameters further illustrate the heightened severity of disease at presentation among patients with DUA-IE. Table 2 shows significantly elevated inflammatory markers, namely white blood cell counts and C-reactive protein levels, in DUA-IE compared to non-DUA-IE. This exaggerated inflammatory response likely reflects the virulence of organisms commonly associated with injection drug use and delays in seeking medical care [10]. The microbiological results reinforce this interpretation: *S. aureus* accounted for over half of the DUA-IE cases and was significantly more common than that in non-DUA-IE. The predominance of *S. aureus*, known for its rapid vegetation formation, endothelial invasion, and embolic potential, provides a compelling explanation for the aggressive clinical course observed in DUA-IE cases.

The exclusive occurrence of Gram-negative organisms, such as *K. pneumoniae,* in DUA-IE further supports the conclusion that injection practices directly shape pathogen profiles [11].

The echocardiographic profile of our cohort underscores the markedly aggressive nature of DUAIE. Vegetations were significantly larger in the DUA-IE group, a feature consistently associated with a higher embolic risk and the need for earlier surgical evaluation [12,13]. Larger vegetations may stem from the high prevalence of *S. aureus* bacteremia, rapid bacterial proliferation, and delays in seeking medical care among individuals who inject drugs [14]. The higher frequency of severe valvular regurgitation among DUA-IE patients highlights the extensive leaflet destruction and rapid hemodynamic deterioration typical of *S. aureus*–driven infections [14]. Furthermore, periannular complications, including abscesses and leaflet perforation, were nearly twice as common in the DUA-IE group, providing an anatomical rationale for the greater surgical burden observed in these patients. The valve-specific analysis of regurgitation severity (Table 4) further refines this profile, showing that severe tricuspid regurgitation is disproportionately represented in DUA-IE, consistent with predominant right-sided involvement, whereas severe aortic and mitral regurgitation occur at comparable frequencies across groups and are more closely related to surgical selection rather than drug use status.

These structural findings align with the broader pattern of more severe inflammatory responses, more frequent septic complications, and poorer overall outcomes in DUA-IE, emphasizing the need for early imaging, multidisciplinary evaluation, and timely surgical intervention when feasible [15,16]. However, a paradox emerged when analyzing the surgical data. Although right-sided (particularly tricuspid) IE is well recognized as the hallmark of injection drug use, the majority of DUA-IE patients who underwent valve surgery required left-sided procedures. Aortic valve replacement accounted for 64.2% of surgeries among DUA-IE patients, while tricuspid valve replacement occurred in only 10.7% of patients, which was lower than that in the non-DUA-IE group (23.6%). This discrepancy reflects not a deviation from the expected pathophysiology but rather the intrinsic nature of the surgical decision-making in IE. This distinction between epidemiological valve involvement and surgical valve distribution reflects disease severity rather than differences in underlying valve pathology.

Right-sided IE, particularly isolated tricuspid valve involvement, frequently responds well to antimicrobial therapy [17]. Even large tricuspid vegetations may resolve with medical management, and septic pulmonary emboli, although common, are seldom absolute indications for surgery [18]. Conversely, left-sided IE, whether in DUA-IE or non-DUA-IE, tends to cause structural valve destruction, severe regurgitation, heart failure, persistent bacteremia, and periannular complications, all of which more commonly necessitate operative interventions [18]. Thus, surgical cohorts inherently reflect a concentration of left-sided pathology, even within populations in which right-sided infection predominates. This “surgical paradox” has been described in multiple international studies and is strongly supported by the distribution observed in our cohort [18,19].

The surgical procedures performed in this study further highlight the advanced disease in patients with DUAIE. Most interventions involve valve replacement rather than repair due to extensive leaflet destruction, perforation, or annular involvement. Aortic valve procedures frequently require radical debridement, and in some cases, root reconstruction. Mitral valve surgery is primarily performed for severe leaflet disruption or subvalvular structure failure [20]. Tricuspid operations, although less common, typically involve the removal of bulky vegetations and replacement when leaflet integrity is inadequate for repair [20,21]. Prosthesis selection was influenced by patient age and anticipated adherence to anticoagulant therapy. Mechanical valves offer greater durability for younger patients but require strict anticoagulation, posing challenges for individuals with unstable living conditions or ongoing substance-use disorders [21,22]. Bioprosthetic valves, although less durable, are often selected to reduce the risks associated with anticoagulant management [22].

The comparative analysis of complications and outcomes (Table 7) highlights the disproportionate clinical severity of DUA-IE, reflected by significantly higher rates of emergency surgery, prolonged hospitalization, discharge against medical advice, and in-hospital mortality despite the younger age of affected patients.

The clinical outcomes further underscore the severity of DUA-IE. As shown in Table 8, patients with DUA-IE experienced significantly longer hospitalizations, reflecting both the complexity of the infection and the need for prolonged postoperative care [22,23].

They were less frequently discharged home and more likely to leave the hospital against medical advice, a pattern repeatedly associated with inadequate completion of antibiotic therapy, recurrent infection, and increased mortality. Multiple interacting factors likely contribute to this disparity, including virulent pathogens, severe inflammation, immunosuppression due to HIV or HCV coinfection, and socioeconomic barriers to treatment.

The burden of infective endocarditis–related complications observed in this cohort highlights the aggressive nature of DUA-IE [23]. Beyond mortality, patients with DUA-IE experience high rates of persistent bacteremia, sepsis, embolic phenomena, heart failure, and periannular extension, all of which contribute substantially to morbidity and surgical complexity [24]. These complications reflect both the virulence of the causative pathogens, particularly *S. aureus*, and delays in presentation or treatment adherence [24]. Therefore, comprehensive screening for complications using echocardiography and targeted imaging is essential for timely surgical referral and outcome optimization in this population [25].

Antimicrobial management is a central challenge in the care of patients with DUA-IE. Current European guidelines support prolonged intravenous antibiotic therapy and, in selected cases, outpatient parenteral antibiotic therapy for clinically stable patients [24,25]. In our cohort, inpatient intravenous therapy remained the predominant treatment approach, particularly among patients with DUA-IE owing to the high rates of persistent infection, frequent complications, discharge against medical advice, and social vulnerability. Although outpatient antibiotic therapy may reduce hospitalization duration and healthcare costs, its implementation in patients with active substance use is limited by concerns regarding adherence, safe venous access, and the availability of structured outpatient monitoring [25]. These real-world constraints highlight the gap between guideline recommendations and practical feasibility and underscore the need for integrated addiction and infectious disease services to support safer transitions to outpatient care for patients with OUD.

Taken together, these findings characterize DUA-IE as a distinct and highly complex syndrome. Despite affecting younger individuals, DUA-IE is associated with more aggressive infections, higher inflammatory responses, greater microbiological virulence, more severe complications, and significantly worse outcomes [25]. The disproportionate surgical burden of left-sided disease in DUA-IE reflects the natural history of IE severity, rather than contradicting the established epidemiology [26]. The need for an integrated management model incorporating infectious disease expertise, cardiac surgery, addiction medicine, psychosocial services, and harm-reduction strategies is evident throughout the data [26].

The antimicrobial susceptibility patterns observed in this cohort further illuminate the clinical differences between DUA-IE and non-DUA-IE cases [27]. Although the overall resistance profiles were broadly comparable, notable trends were observed. Patients with DUA-IE exhibited a higher proportion of MSSA than those without DUA-IE; however, the latter group demonstrated significantly higher rates of MRSA, suggesting distinct epidemiological exposures and patterns of healthcare contact. Conversely, DUA-IE showed slightly elevated methicillin resistance within *S. aureus* isolates compared with community expectations, as well as marginally higher rates of resistance to aminoglycosides among *E. faecalis*. These findings likely reflect repeated bacteremia episodes, intermittent healthcare exposure, and inconsistent antibiotic access among individuals who inject drugs, all of which exert selective pressure on circulating organisms [28,29]. The exclusive presence of ESBL-producing *K. pneumoniae* within the DUA-IE group further underscores the complex interactions between polysubstance use, environmental contamination, and polymicrobial inoculation risk during injection practices [30]. Despite these differences, most isolates remained susceptible to guideline-recommended agents, and prompt initiation of empiric therapy with anti-staphylococcal β-lactams or glycopeptides remained appropriate for both populations [31,32]. Ultimately, the susceptibility data emphasize that while resistance patterns alone do not fully account for the poorer outcomes observed in DUA-IE, early broad-spectrum coverage and rapid tailoring of therapy remain essential for mitigating the effects of aggressive pathogens, extensive comorbidities, and delays in care characteristic of this high-risk population [33].

This study had some limitations. Its retrospective, single-center design restricts the generalizability of the findings and reflects the characteristics of a specific regional population sample. Some clinically relevant variables, including detailed embolic complications, comprehensive hemato chemical parameters, and certain echocardiographic features, were not consistently available, limiting the precision of subgroup analyses. Long-term follow-up data were not collected, preventing the assessment of recurrent infective endocarditis, prosthetic valve durability, or post-discharge mortality. Surgical findings are subject to inherent selection bias, as patients with right-sided disease who improved with medical therapy were unlikely to undergo surgery and were, therefore, underrepresented in operative cohorts. Information on substance use patterns, access to harm-reduction services, and engagement with addiction treatment was also unavailable, potentially influencing the outcomes. The study also lacked multivariable regression due to sample size constraints, which limited the causal inference. Although part of the study period overlapped with the COVID-19 pandemic, a separate pre-pandemic versus pandemic subgroup analysis was not performed because of the limited sample size within each year and the exploratory nature of the study. Annual trends are presented in Figure 1 to illustrate temporal variations. Finally, microbiological analyses were based on conventional culture methods without molecular characterization, limiting the insights into pathogen virulence. Despite these constraints, this study provides valuable evidence of the clinical and surgical burden of drug–use–associated infective endocarditis in a region where data are limited.

## 5. Conclusions

In this six-year cohort study, drug use–associated IE was associated with younger age but more aggressive disease, higher complication rates, greater surgical burden, and increased in-hospital mortality compared with non–drug use–associated IE. Despite frequent right-sided involvement, left-sided valve disease predominated among surgically treated patients with DUA-IE, reflecting greater hemodynamic severity. The high prevalence of infectious comorbidities and frequent discharge against medical advice further complicates the management and worsens the outcomes. These findings underscore the need for early multidisciplinary evaluation and integrated care models that address both the severity of infection and substance use disorders.

## Figures and Tables

**Table 1:** Baseline characteristics of patients with infective endocarditis according to drug use status.

Variable	All IE (n = 189)	DUA-IE (n = 43)	Non-DUA-IE (n = 146)	*p*-Value
Age, years, median (IQR)	60 (50–78)	36 (24–41)	62 (53–89)	<0.002
Male sex, n (%)	121 (64.0)	27 (62.8)	94 (64.4)	<0.001
Female sex, n (%)	68 (36.0)	16 (37.2)	52 (35.6)	<0.001
IE hospitalizations, n (%)	189 (100)	43 (22.8)	146 (77.2)	<0.001

**Table 2:** Hematochemical parameters in patients with DUA-IE and non-DUA-IE.

Parameter	DUA-IE (n = 43)	Non-DUA-IE (n = 146)	*p*-Value
White blood cell count (×10^3^/µL)	15.2 ± 4.5	12.6 ± 3.8	0.03
C-reactive protein (mg/L)	120.5 ± 42.1	95.3 ± 40.8	0.02
Creatinine (mg/dL)	1.2 ± 0.5	1.0 ± 0.3	0.15
Hemoglobin (g/dL)	11.3 ± 2.1	12.0 ± 2.3	0.07

**Table 3:** Distribution of etiological agents in DUA-IE and non-DUA-IE.

Pathogen	DUA-IE, n (%)	Non-DUA-IE, n (%)	*p*-Value
*Staphylococcus aureus*	24 (55.9)	63 (43.1)	<0.003
*Enterococcus faecalis*	7 (16.4)	41 (28.2)	<0.001
*Streptococcus mitis*	6 (13.9)	32 (21.9)	<0.001
*Staphylococcus epidermidis*	3 (6.9)	10 (6.8)	0.99
*Klebsiella pneumoniae*	3 (6.9)	0	<0.003

**Table 4:** Echocardiographic Characteristics in DUA-IE and Non-DUA-IE Patients.

Echocardiographic Parameter	DUA-IE (n = 43)	Non-DUA-IE (n = 146)	*p*-Value
Median vegetation size, mm (IQR)	16 (12–22)	12 (8–17)	<0.001
Severe aortic regurgitation, n (%)	11 (25.6%)	42 (28.8%)	0.85
Severe mitral regurgitation, n (%)	8 (18.6%)	34 (23.3%)	0.68
Severe tricuspid regurgitation, n (%)	12 (27.9%)	9 (6.2%)	<0.001
Abscess or leaflet perforation, n (%)	8 (18.4%)	16 (11.3%)	0.20

**Table 5:** Characteristics of patients who underwent valve surgery between 2017 and 2022.

Variable	All Surgical IE (n = 83)	DUA-IE (n = 28)	Non-DUA-IE (n = 55)	*p*-Value
Age, years, median (IQR)	56 (37–62)	31 (22–39)	59 (47–64)	<0.002
Male sex, n (%)	51 (64.0)	20 (71.4)	31 (56.3)	<0.001
Female sex, n (%)	32 (36.0)	8 (28.6)	24 (43.6)	<0.002
Aortic valve surgery, n (%)	38 (45.8)	18 (64.2)	20 (36.3)	<0.002
Mitral valve surgery, n (%)	29 (34.9)	7 (25.0)	22 (40.1)	<0.002
Tricuspid valve surgery, n (%)	16 (19.3)	3 (10.7)	13 (23.6)	<0.002

**Table 6:** Comorbidities in patients with DUA-IE and non-DUA-IE.

Comorbidities	DUA-IE (n = 43), n (%)	Non-DUA-IE (n = 146), n (%)	*p*-Value
Hepatocellular carcinoma	2 (4.6)	1 (0.6)	<0.03
HIV infection	28 (65.1)	2 (1.37)	<0.01
Hepatitis C	11 (25.5)	11 (7.5)	<0.003
Hepatitis B	2 (4.6)	0	<0.02
Chronic renal failure	1 (2.3)	14 (9.5)	<0.05
Hypertension	3 (6.9)	78 (53.4)	<0.001
Chronic alcoholism	14 (32.5)	0	<0.003
Esophageal varices	2 (4.6)	0	<0.03
Cellulitis	16 (37.2)	0	<0.001
Necrotizing fasciitis	1 (2.3)	0	<0.003
AIDS	2 (4.6)	0	<0.002
Stroke	4 (9.3)	40 (27.3)	<0.001
Syphilis	1 (2.3)	0	<0.03
Urinary tract infections	8 (18.6)	17 (11.6)	<0.002
Pneumonia	4 (9.3)	4 (2.7)	<0.006

**Table 7:** Infective endocarditis–related complications and in-hospital outcomes according to drug use status.

Complication	DUA-IE (n = 43)	Non–DUA-IE (n = 146)	*p*-Value
Periannular complications (abscess/perforation)	8 (18.4%)	16 (11.3%)	0.20
Severe valvular regurgitation	19 (45.3%)	51 (34.7%)	0.29
Median vegetation size, mm (IQR)	16 (12–22)	12 (8–17)	<0.001
Stroke	4 (9.3%)	40 (27.3%)	0.013
Emergency valve surgery	28 (65.1%)	55 (37.7%)	0.002
Discharge against medical advice	8 (18.6%)	6 (9.5%)	0.004
In-hospital mortality	4 (9.3%)	2 (1.37%)	0.025
Median hospital stay, days (IQR)	17 (9–24)	9 (7–21)	<0.001

**Table 8:** Hospital stay and in-hospital outcomes according to drug use status and valve surgery.

Outcome	All IE (n = 189)	DUA-IE (n = 43)	Non-DUA-IE (n = 146)	*p*-Value
Hospital stay, days, median (IQR)	11 (5–20)	17 (9–24)	9 (7–21)	<0.001
Discharged home, n (%)	127 (67.2)	31 (53.4)	96 (75.6)	<0.002
Rehabilitation center, n (%)	6 (3.1)	6 (13.9)	0	–
Nursing facility, n (%)	17 (9.0)	1 (2.3)	16 (9.0)	<0.02
In-hospital death, n (%)	6 (3.1)	4 (9.3)	2 (1.37)	<0.02
Unknown status, n (%)	22 (9.0)	1 (2.3)	21 (14.3)	<0.01
Discharge against medical advice, n (%)	14 (7.4)	8 (18.6)	6 (9.5)	–

## Data Availability

The data presented in this study are available on request from the corresponding author, the data are not publicly available due to privacy or ethical restrictions.
